# CircAHNAK1 inhibits proliferation and metastasis of triple-negative breast cancer by modulating miR-421 and RASA1

**DOI:** 10.18632/aging.102539

**Published:** 2019-12-19

**Authors:** Weikai Xiao, Shaoquan Zheng, Yutian Zou, Anli Yang, Xinhua Xie, Hailin Tang, Xiaoming Xie

**Affiliations:** 1 Department of Breast Oncology, Sun Yat-sen University Cancer Center, State Key Laboratory of Oncology in South China, Collaborative Innovation Center for Cancer Medicine, Guangzhou 510060, People’s Republic of China

**Keywords:** biomarker, triple negative breast cancer, circAHNAK1, miR-421, RASA1

## Abstract

Background: There is increasing evidence that circular RNAs (circRNAs) participate in regulating cancer progression. However, the function and potential molecular mechanisms of circRNA in triple negative breast cancer (TNBC) are currently largely unclear.

Results: We found that circAHNAK1 was significantly down-regulated in TNBC, and its expression was negatively associated with RFS and OS. Overexpression of circAHNAK1 can inhibit TNBC proliferation, migration and invasion in vitro. In vivo studies confirmed that circAHNAK1 inhibited TNBC tumor growth and metastasis. Mechanistic analysis indicated that circAHNAK1 acted as a miR-421 ceRNA (competitive endogenous RNA) to attenuate the inhibitory effect of miR-421 on its target gene RASA1.

Conclusions: In conclusion, CircAHNAK1 inhibits proliferation and metastasis of TNBC by modulating miR-421 and RASA1.

Methods: CircRNA microarrays were used to screen for differential circRNA expression profiles. qRT-PCR was used to detect the expression levels of circRNAs. The effect of circAHNAK1 on recurrence -free survival (RFS) and overall survival (OS) in patients with TNBC was subsequently analyzed. The role of circAHNKA1 in the progression of TNBC was further evaluated by multiple in vivo and in vitro assays. Finally, we focused on the regulation of circAHNAK1 on miR-421 and its targeted gene RASA1 in TNBC.

## INTRODUCTION

Triple-negative breast cancer (TNBC) is one of the most deadly types of breast cancer. TNBC featured with highly invasive and highly metastatic, resulting in a poor prognosis [1]. The 5-year mortality rate for TNBC is still relatively high due to difficulties in curbing recurrence and metastasis and poor response to various treatments [2]. Although recent research on non-TNBC treatment has made great progress, the treatment of TNBC is still limited [3]. Non-coding RNA plays an important regulatory role in the biological properties of cancer and is also considered a potential therapeutic target for TNBC. Some endogenous non-coding RNAs such as circRNA are closely related to tumor cell proliferation, apoptosis and metastasis by regulating a large number of signaling pathways in cancer [4, 5]. In particular, circRNA can act as a microRNA sponge to regulate gene expression [6, 7]. The study of dysregulated circRNAs and their function in TNBC has attracted increasing attention [8–10].

Here, we reanalyzed the circRNA expression profiles of the TNBC tissue and the matched para-cancer normal tissue we performed in our previous study [11]. We found a circRNA derived from the AHNAK gene and named it circAHNAK1, which was significantly lower in TNBC tissues than in normal tissues, and that low expression of cirAHNAK1 was associated with shortened survival. Subsequently, we conducted a variety of experiments to further confirm that circAHNAK1 played an important role in the development of TNBC. In vitro, down-regulation of circAHNAK1 promoted the proliferation and invasion of TNBC cells. Decreased expression of cirAHNAK1 in TNBC cells increased tumor proliferation, migration and invasion. In terms of mechanism, cirAHNAK1 may promote the expression of the tumor suppressor gene RASA1 through sponging miR-421, thereby exerting its regulatory function in TNBC. Therefore, this study suggests that circAHNAK1 is a prognostic biomarker for TNBC and a therapeutic target that can be used for TNBC patient.

## RESULTS

### CircANNAK1 is down-regulated in TNBC patients and significantly affects the prognosis of TNBC

**Figure 1:**
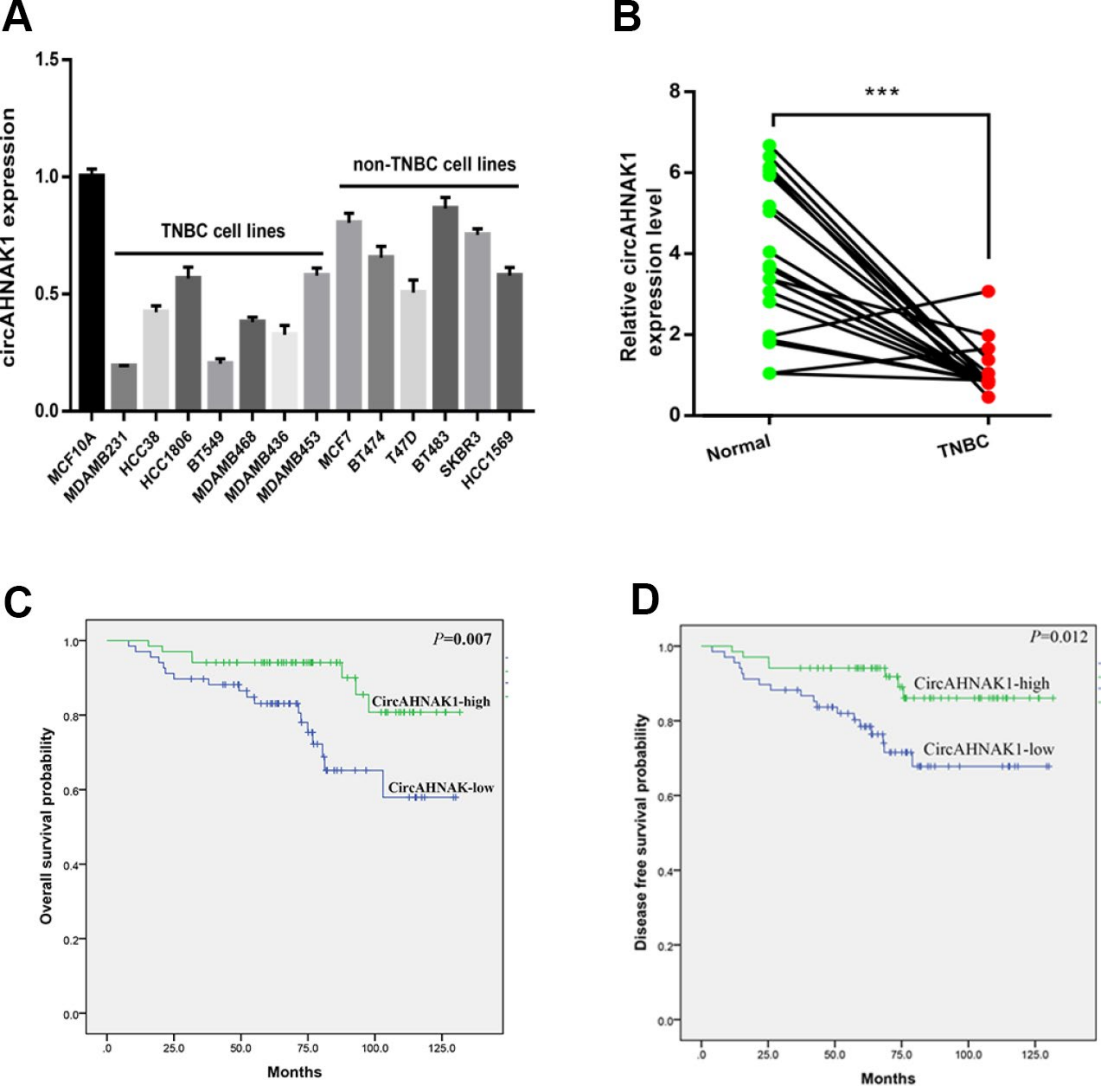
**circANNAK1 is down-regulated and associated with malignant progression and poor prognosis of TNBC.** (**A**) Expression of circAHNAK1 in breast cancer cell lines. (**B**) Expression of circAHNAK1 in breast cancer tissues and normal adjacent tissues. (**C**, **D**)The effect of circAHNAK1 expression on OS and DFS in patients with TNBC. *** *P* <0.001.

To study the function and role of circRNAs in TNBC, the circRNA microarrays performed in our previous study were reanalyzed by us. qRT-PCR was used to verify the expression level of one of the most obvious down-regulated circRNAs, hsa_circ: chr11:62297840-62298224(hsa_circ_0000320), in TNBC tissues and cell lines. According to the information provided by the human reference genome (GRCh37/hg19), hsa_circ_0000320 is derived from the AHNAK gene according to predictions. Therefore, circRNA hsa_circ_0000320 is named “circAHNAK1” by us. In order to verify the circular structure of circAHNAK1, we performed RNase R digestion test and the actinomycin D test. Resistance to RNase R exonuclease further confirmed that the RNA is a circular structure (Supplementary Figure 1A). In addition, actinomycin D analysis indicated that the half-life of the circular RNA circAHNAK1 was longer, indicating the stability of circAHNAK1 (Supplementary Figure 1B). According to the results of qRT-PCR, the expression level of circAHNAK1 in the TNBC cell line was significantly lower than that of the Non-TNBC cell line (Figure 1A). Then, we evaluated its expression in TNBC tissue and adjacent normal tissues. Expression of circAHNAK1 was also found to be significantly down-regulated in TNBC tissues (Figure 1B). ROC analysis suggested that circAHNAK1 can be used as a diagnostic indicator for distinguishing between TNBC and normal breast tissue (Supplementary Figure 1C). Our result also suggested that circRNA is significantly down-regulated in stage III–IV TNBC tissues compared to stage I–II (Supplementary Figure 1D).

To investigate the clinical significance of circAHNAK1 in TNBC, we analyzed the expression of circAHNAK1 in 136 TNBC patients. We used the median value of the expression as the cutoff value and classified all TNBC patients as “circAHNAK1-high” and “circAHNAK1-low “ groups. We found that circAHNAK1 expression was negatively correlated with larger tumors(*P*=0.045), positive lymph node metastasis(*P*=0.001), and late TNM stage (*P*<0.001, Table 1), suggesting that circAHNAK1 may play an important role in the malignant progression of TNBC. Subsequently, we performed survival analysis and found that low expression of circAHNAK1 in TNBC patients predicted poor OS and DFS (Figure 1C and 1D).

**Table 1:** Relationship between circAHNAK1 and clinical-pathological features of TNBC.

**Variables**	**Cases(n=136)**	**circAHNAK1**		***P* value**
		**Low No.(N=68)**	**High No.(N=68)**	
**Age(years)**				
<40	35	15(42.9%)	20(57.1%)	0.393
40-60	87	44(50.6%)	43(49.4%)	
>60	14	9(64.3%)	5(35.7%)	
**Menopause**				
No	82	41(50.0%)	41(50.0%)	1.000
Yes	54	27(50.0%)	27(50.0%)	
**T stage**				
T1-T2	122	57(46.7%)	65(53.3%)	0.045^*^
T3-T4	14	11(78.6%)	3(21.4%)	
**N stage**				
N0	75	27(36.0%)	48(64.0%)	0.001^*^
N1-3	61	41(67.2%)	20(32.8%)	
**TNM stage**				
I-II	107	43(40.2%)	64(59.8%)	<0.001^*^
III-IV	29	25(86.2%)	4(13.8%)	

^*^P < 0.05, statistically significant

### Overexpression of circAHNAK1 inhibits TNBC proliferation and metastasis

**Figure 2:**
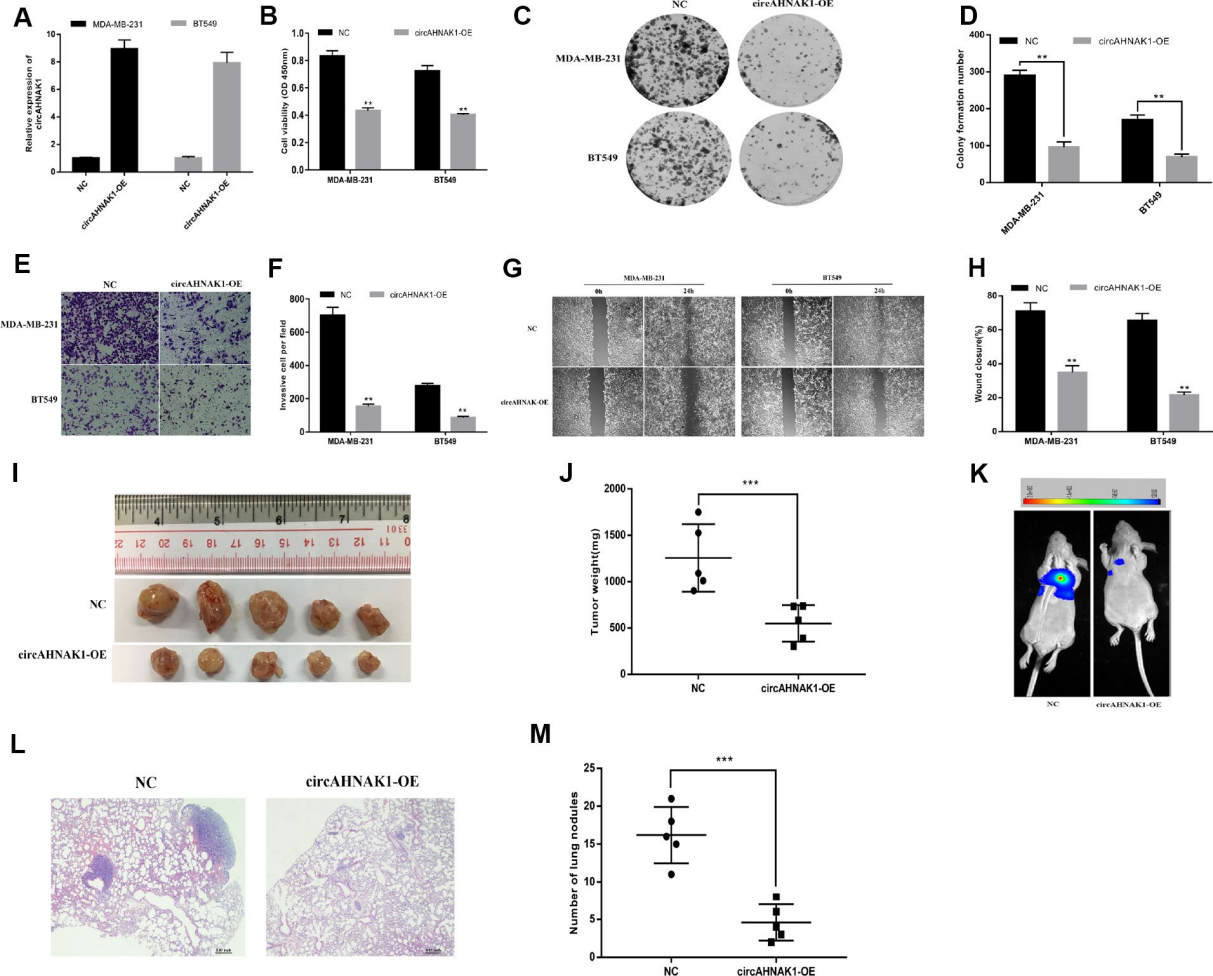
**Overexpression of circAHNAK1 inhibits proliferation and metastasis of TNBC.** (**A**)Successfully established two breast cancer cell lines that overexpress circAHNAK1; (**B**) CCK-8 assay to evaluate the effect of circAHNAK1 on cell proliferation; (**C**) Colony formation assay to evaluate the effect of circAHNAK1 on cell colony forming ability; (**D**) Number of clones quantified by ImageJ; (**E**) Transwell invasion assay to evaluate the effect of circAHNAK1 on cell invasion; (**F**) ImageJ quantifies the number of invading cells; (**G**) Wound healing assay evaluates the effect of circAHNAK1 on wound closure; (**H**) ImageJ quantifies the extent of wound healing; (**I**) Xenograft model to evaluate the effect of circAHNAK1 on tumor proliferation in vivo; (**J**) Effect of circAHNAK1 on proliferation in vivo by tumor weight; (**K**) Representative images of luciferase signaling to assess the effects of circAHNAK1 on lung metastasis in vivo; (**L**) Representative images of HE staining of lung metastatic nodule sections; (**M**) Quantification of the number of lung metastatic nodules.

To investigate the function of circAHNAK1 in TNBC, we transfected the overexpression vector of circAHNAK1 in MDA-MB-231 and BT-549 cells, for subsequent studies (Figure 2A). CCK-8 assay suggested that overexpression of circAHNAK1 significantly inhibits proliferation of TNBC cells (Figure 2B). Overexpression of circAHNAK1 also inhibited the colony formation (Figure 2C and 2D). The invasive ability of TNBC cells after circAHNAK1 overexpression was found to be significantly reduced according to the invasion assay (Figure 2E and 2F). Migration assays suggested that circAHNAK1 overexpression significantly inhibited migration potential compared to controls (Figures 2G and 2H). Subsequently, we established a mouse xenograft model to investigate the role of circAHNAK1 in vivo. Overexpression of circAHNAK1 significantly reduced tumor volume and weight (Figure 2I and 2J). Moreover, the size and number of lung metastases were also significantly inhibited by overexpression of circAHNAK1 (Figure 2K–2M), indicating circAHNAK1 can inhibits the malignant progression of TNBC cells.

### circAHNAK1 sponges miR-421 in TNBC

**Figure 3:**
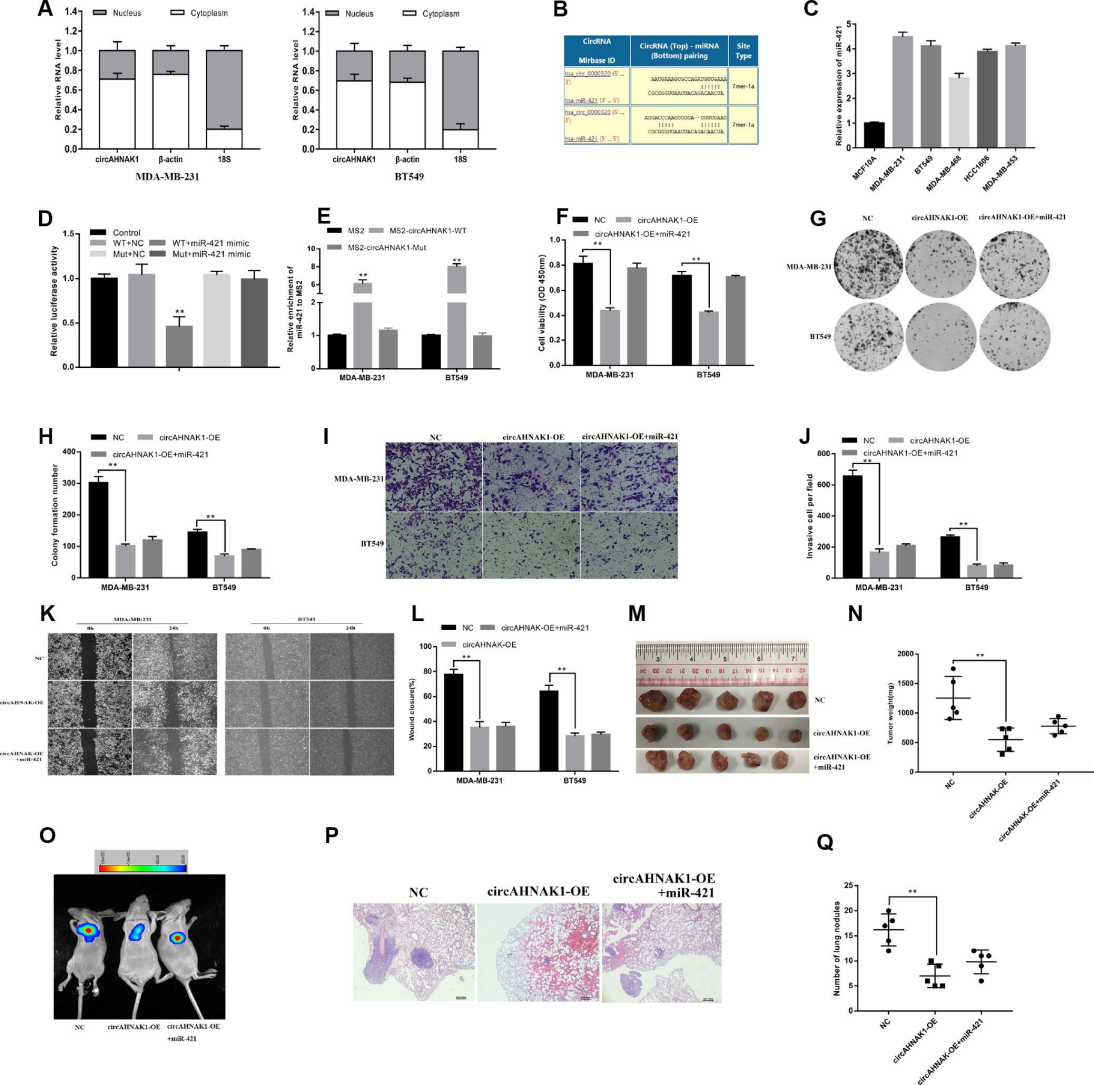
**circAHNAK1 can act as a sponge for miR-421.** (**A**) The expression levels of nuclear control (18S), cytoplasmic control (β-actin) and circAHNAK1 were detected; (**B**) Prediction of the binding site of miR-421 in the circAHNAK1 sequence; (**C**) The expression of miR-421 in TNBC cell line; (**D**) Luciferase assay of cells co-transfected with the miR-421 mimics and the circ-AHNAK1 wild type or mutant luciferase reporter; (**E**) GFP-MS2-RIP assay detects enrichment of miR-421after MS2bs-circAHNAK1-WT, MS2bs-circAHNAK1-Mut or control transfection, respectively; (**F**) Effect of miR-421 mimics transfection on proliferation of circAHNAK1 overexpressing cells by CCK-8; (**G**) Effect of miR-421 mimics transfection on circAHNAK1 overexpressing cells by clone formation assay; (**H**) Quantitative clone formation by ImageJ software; (**I**) Transwell invasion assay assessed the impact on cell invasion;(**J**) ImageJ software quantifies the number of invading cells;(**K**)Wound healing assay for detecting changes in cell migration ability; (**L**) ImageJ software quantifies the extent of wound closure; (**M**) Xenograft model to evaluate tumor proliferation in vivo; (**N**) Comparison of tumor weight; (**O**)Representative images of luciferase signaling of lung metastasis in vivo; (**P**) Representative images of HE staining of lung metastatic nodule sections; (**Q**) Quantification of the number of lung metastatic nodules.

We found that circAHNAK1 is mainly distributed in the cytoplasm in cells (Figure 3A), suggesting that it may have a role through sponging miRNA. In order to predict potential miRNAs that bind to circAHNAK1, Circular RNA Interactome was utilized in this study (https://circinteractome.nia.nih.gov/). We found two binding sites for miR-421 in the circAHNAK1 sequence (Figure 3B). Using qRT-PCR, we found that miR-421 was upregulated in TNBC cell lines (Figure 3C). We then observed that co-transfection of miR-421 mimics and the wild-type vector significantly reduced luciferase activity, but no similar phenomenon was observed for transfection of the mutant luciferase reporter (Figure 3D). To confirm that circAHNAK1 interacts directly with miR-421, we conducted a GFP-MS2-RIP assay. The results showed that the enrichment of miR-421 was mainly in the MS2-circAHNAK1-WT group, but not the MS2-circAHNAK1-Mut group (Figure 3E) compared to the negative control. This result further indicated that circAHNAK1 could directly bind to miR-421

To investigate whether circAHNAK1 exerts its effects via sponge miR-421, we conducted multiple in vivo and in vitro studies. CCK-8 and colony formation assays showed that miR-421 mimics could partially reverse the inhibition of cell proliferation induced by circAHNAK1 overexpression (Figure 3F–3H). Invasion and migration assays showed that treatment with miR-421 mimics also partially reverse the decreased cell migration capacity caused by circAHNAK1 overexpression (Figure 3I–3L). Experiments using a xenograft mouse model showed that treatment with miR-421 mimics also partially reverse the reduction in tumor growth and lung metastasis after circAHNAK1 overexpression (Figure 3M–3Q). All these findings indicate that circAHNAK1 plays a role in TNBC by sponging miR-421. Together, these results provide evidence that circAHNAK1 functions in TNBC via sponging miR-421.

### circAHNAK1 sponges miR-421 to regulate RASA1 expression

**Figure 4:**
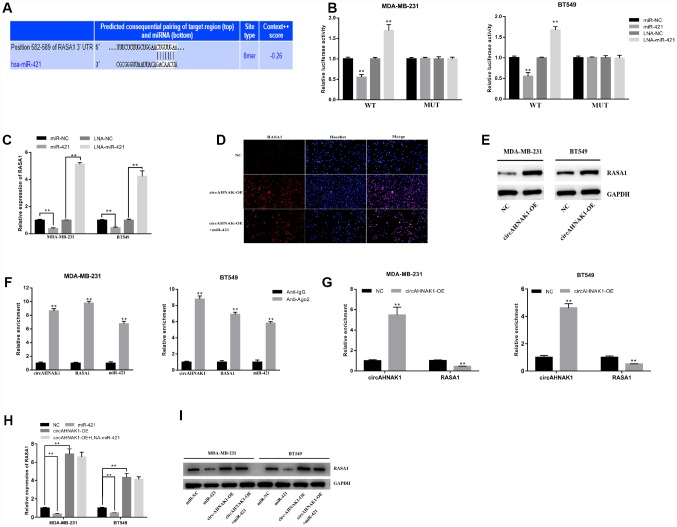
**circAHNAK1 acts as a ceRNA to regulate RASA1.** (**A**)Predicting the binding site of miR-421 in the RASA1-3′UTR region by TargetScan;(**B**) Luciferase assay after transfection with miR-421 mimics or inhibitor (**C**) Detection of RASA1 expression after miR-421 transfection by qRT-PCR; (**D**) Detection of RASA1 expression after transfection by immunofluorescence; (**E**)Detection of RASA1 expression after transfection by western blots. (**F**)Ago2-RIP assay showed enrichment of circAHNAK1, RASA1 and miR-421 on Ago2; (**G**) Ago2-RIP assay showed the effect of circAHNAK1 overexpression on RASA1 enrichment; (**H**) qRT-PCR detected RASA1 expression after transfection of circAHNAK1 and/or miR-421; (**I**)Western blots detected RASA1 expression after transfection of circAHNAK1 and/or miR-421;

To investigate whether circAHNAK1 regulates its downstream target genes via sponging miR-421, we searched potential target genes for miR-421 in TargetScan and predicted RASA1 (Figure 4A). We then found that luciferase activity was decreased after co-transfection of miR-421 mimics and wild-type luciferase by luciferase reporter assay, whereas co-transfection with mutant luciferase reporter had no similar effect (Figure 4B). In addition, after co-transfection of the miR-421 inhibitor and wild-type luciferase reporter, we observed a significant increase in luciferase activity(Figure 4B). Furthermore, miR-421 mimics inhibited the expression of RASA1, while the miR-421 inhibitor increased the expression of RASA1 (Figure 4C), indicating that RASA1 could be regulated by miR-421.

In addition, circAHNAK1 overexpression upregulated the expression of RASA1 by both immunofluorescence and Western blot (Figure 4D, 4E). The results of correlation analysis suggested that there is a positive correlation between the expression of circAHNAK1 and RASA1 in TNBC (r=0.4884, P<0.01; Supplementary Figure 1E). The Anti-Ago2-RIP assay indicated that circAHNAK1, RASA1 and miR-421 were predominantly enriched to Ago2 compared to control immunoglobulin G (IgG) antibodies (Figure 4F), indicating that circAHNAK1 and RASA1 were recruited to Ago2-related RNA-induced Silencing Complex (RISC), where they can interact with miR-421. Then, we observed that overexpression of circAHNAK1 increased circAHNAK1 enrichment in Ago2-RIPs and reduced RASA1 enrichment in Ago2-RIPs (Figure 4G), indicating that circAHNAK1 acts as a ceRNA and competes with RASA1 for binding to miRNA. Furthermore, transfection with miR-421 resulted in reduced expression of RASA1, whereas overexpression of circAHNAK1 rescued the reduction (Figure 4H and 4I), indicating that circAHNAK1 could promote RASA1 expression by sponging miR-421.

### RASA1 is negatively correlated with poor survival of TNBC

**Figure 5:**
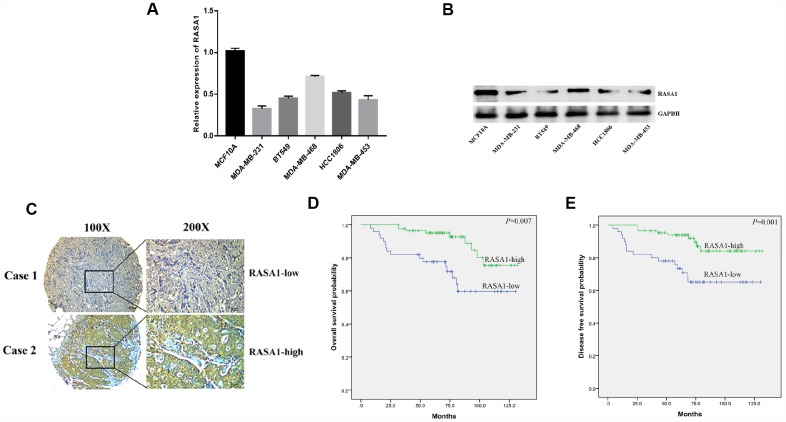
**RASA1 is down-regulated and associated with poor prognosis of TNBC.** (**A**) qRT-PCR detected mRNA expression of RASA1 in breast cancer cell lines;(**B**)Western blots detected RASA1 expression in breast cancer cell lines;(**C**) Representative IHC images of high and low RASA1 expression in TNBC tissues;(**D**, **E**)The effect of RASA1 expression on OS and DFS in patients with TNBC.

We then examined the expression of RASA1 in the TNBC cell line and found that RASA1 was lowly expressed in the TNBC cell lines (Figure 5A and 5B). We also studied the clinical impact of RASA1 expression on TNBC patients, demonstrating that low expression of RASA1 predicted later TNM stage and a higher rate of lymph node metastasis (Figure 5C, Table 2), indicating an important role for RASA1 in the malignant progression of TNBC. Survival analysis also indicated that lower RASA1 expression in TNBC predicted poor OS and DFS (Figure 5D and 5E).

**Table 2:** Relationship between RASA1 and clinical-pathological features of TNBC.

**Variables**	**Cases(n=136)**	**RASA1**		***P* value**
		**Low (n=50)**	**High(n=86)**	
**Age (years)**				
<40	35	13(37.1%)	22(62.9%)	0.230
40-60	87	29(33.3%)	58(66.7%)	
>60	14	8(57.1%)	6(42.9%)	
**Menopause status**				
No	82	28(34.1%)	54(65.9%)	0.274
Yes	54	22(40.7%)	32(59.3%)	
**T stage**				
T1-T2	122	39(32.0%)	83(68.0%)	0.001^*^
T3-T4	14	11(78.6%)	3(21.4%)	
**N stage**				
N0	75	15(20.0%)	60(80.0%)	<0.001^*^
N1-3	61	35(57.4%)	26(42.6%)	
**TNM stage**				
I-II	107	29(27.1%)	78(72.9%)	<0.001^*^
III-IV	29	21(72.4%)	8(27.6%)	

^*^P < 0.05, statistically significant

## DISCUSSION

Circular RNAs are a new class of non-coding RNAs that are gaining more and more attention in the regulation of human cancers. In the current study, we evaluated the function of circ-AHNAK1, which is a novel circRNA derived from the AHNAK locus. We found that circAHNAK1 is an important circRNA that is frequently down-regulated in TNBC tissues, whereas low expression of circAHNAK is positively correlated with malignant progression and poor survival in TNBC patients. Secondly, through mechanism studies, we found that overexpression of circAHNAK1 increased the sponge of miR-421 and up-regulated the expression of the tumor suppressor gene RASA1, thereby inhibiting the proliferation and metastasis of TNBC cells. In view of these findings, circAHNAK1 may be used as a potential biomarker or therapeutic target for human TNBC.

Functionally, recent studies have shown that circRNA molecules are rich in microRNA (miRNA) binding sites and act as miRNA sponges in cells. circPRKCI adsorbed miR-545 and miR-589 by “sponge action” and relieveed its target inhibition, leading to up-regulation of the cancer-promoting transcription factor E2F7 [12]. In liver cancer, circMAT2B could competitively adsorb miR-338-3p by sponge-like, and then regulated the key metabolic enzyme PKM2 (pyruvate kinase M2), which promoted the progression of tumor malignancy [13]. Also in liver cancer, the circular RNA circMTO1 acted as a sponge for microRNA-9 to inhibit the progression of hepatocellular carcinoma [14]. PRMT5 circular RNA induced the epithelial-mesenchymal transition of bladder urothelial carcinoma through spongy miR-30c to promote its metastasis [13]. In gastric cancer, circNRIP1 sponging microRNA-149-5p regulated the AKT1/mTOR pathway to promote progression through [14].

miR-421 was previously discovered to promote tumor progression, which is remarkedly upregulated in a variety of cancer types. In hepatocellular carcinoma (HCC), down-regulation of FXR by miR-421 promoted proliferation, migration and invasion of HCC cells [15]. In gastric cancer, miR-421 is significantly upregulated and promoted the growth of gastric cancer cells [16]. miR-421 was found to be upregulated in breast cancer tissues and inhibits apoptosis by inhibiting caspase-10 expression [17]. The up-regulated expression of miR-421 is associated with poor prognosis in non-small cell lung cancer [18]. Overexpression of miR-421 leaded to down-regulation of ATM and is associated with poor prognosis of breast cancer [19]. In prostatic cancer, miR-421 was also found to inhibit ATM expression and promote prostate cancer metastasis and treatment resistance [20].

RASA1 is a member of the RAS GTPase Activating Protein (RAS-GAP) family. The well-known oncoprotein RAS can be inactivated by binding to RAS-GAP members. Some studies have shown that mutation or loss of function of RASA1 leads to activation of the RAS-MAPK cascade in malignant tumors. RASA1 has been identified as a tumor suppressor gene which is underexpressed in multiple malignancies. RASA1 expression is also remarkedly negatively associated with survival outcome in a variety of malignancies. The dysregulation of RASA1 plays an important role in the progression of malignant tumors, so it may become a predictor for prognosis and therapeutic target. Suárez-Cabrera C et al. confirmed by immunohistochemical analysis of human breast tumor tissues that low expression of RASA1 is common in basal (triple negative) and estrogen receptor negative tumors [21]. It has also been found in colorectal cancer that RASA1 mutation or loss of function leads to activation of the RAS-MAPK cascade [21–23]. In non-small cell lung cancer, RASA1 has also been found to be associated with tumor progression and mediated MEK inhibition [24]. In hepatocellular carcinoma, it has also been found that down-regulation of RASA1 promotes angiogenesis under hypoxic conditions [25]. Consistent with previous studies, we also demonstrated that RASA1 is underexpressed in TNBC compared to normal tissues, and the expression of RASA1 is negatively correlated with RFS and OS.

In conclusion, circAHNAK1 is down-regulated in TNBC and its expression is negatively correlated with survival outcome in patients with TNBC. circAHNAK1 regulates TNBC cell proliferation and invasion, and the mechanism may be related to the expression of the tumor suppressor gene RASA1 by sponge miR-421, thereby promoting the progression of TNBC. The circAHNAK1-miR-421-RASA1 axis participates in TNBC progression through a competitive ceRNA mechanism. Therefore, circAHNAK1 can be used as a predictor of TNBC prognosis and a potential molecular target.

## MATERIALS AND METHODS

### Cell culture and transfection

The cell lines used in this study included human normal breast cell (MCF10A), Non-TNBC cells (BT474, T47D, BT483, SKBR3, MCF-7 ,HCC1569) and TNBC cells (HCC38, HCC1806, BT549, MDA-MB-468, MDA-MB-453, MDA-MB-436, MDA-MB-231) were obtained from the American Type Culture Collection. All cell lines were free of mycoplasma infection and identified by STR (short tandem repeat) to ensure cell reliability. Lipofectamine 2000 (Invitrogen, USA) was used for cell transfection. The overexpression vector of circAHNAK1 was synthesized by IGEbio (China). Inhibitors and mimics of miR-421 were obtained from GeneCopoeia (USA).

### qRT-PCR

Extraction of total RNA, separation of cytoplasm and nuclear RNA was performed as described in the previous study [8]. Real-time PCR analysis of mRNA and circRNA was performed using SYBR Premix Ex TaqTM (Takara, Japan) and Bio-Rad CFX96 detection system (USA). Real-time PCR analysis of miRNA was performed using the All-in-OneTM miRNA qRT-PCR detection kit (GeneCopoeia) and the Bio-Rad CFX96 detection system (USA). Primers for detection were obtained from GENEray (Supplementary Table 1).

### RNase R treatment and Actinomycin D assay

RNA extracted from MDA-MB-231 cells was divided into two parts on average: one for RNase R (Epicentre Technologies, USA) and the other for buffer treatment for control. In the experimental group, 2 μg of total RNA was incubated with RNase R (3 U/ug) for 20 minutes at 37 °C. β-actin was used as an internal control.

For Actinomycin D assay, 1 x 10^5^ cells were seeded into 6-well plates and treated with actinomycin D (2 mg/L; Sigma, USA). Subsequently, the treated cells were collected at 8, 16 and 24 hours, respectively, for qRT -PCR analysis of circAHNAK18 and AHNAK mRNA.

### Cell viability assay

1 x 10^3^ breast cancer cells were cultured into well plates (96-well), and 10 μl of CCK-8 solution (Dojindo Laboratories, Japan) was added 48 hours after transfection. Subsequently, the incubation continued for 2 hours at 37°C. Finally, their absorbance at 450 nm was measured by using a microtiter plate reader (Bio-Tek EPOCH2, USA).

### Colony formation assay

1 x 10^3^ MDA-MB-231 and BT-549 cells were cultured into well plates(6-well), and cultured in a cell culture incubator under normal conditions. The culture was terminated after 10 days, and the clone was fixed with anhydrous methanol, followed by staining with crystal violet solution (0.1%, Sigma) for 30 min at 25°C.

### Migration assay

MDM-MB-231 and BT-549 cells were cultured in six-well plates and scraped with a fine end of a 200 μl pipette tip (time 0 hours). Cell migration was photographed using an inverted microscope at 0 and 24 hours after injury.

### Transwell assay

The invasion test was performed in a 24-well Transwell chamber. 8-μm well inserts were coated with 30 μg matrigel (BD Biosciences). 5 x 10 ^4^ breast cancer cells (200 μl of serum-free medium) were added to the coated upper chamber. 700 μl of medium containing 20% FBS (fetal calf serum) was added as a chemoattractant to the lower chamber. After an appropriate time in an incubator at 37 °C, the cells migrated through the filter were fixed with methanol, stained with 0.5% crystal violet, and counted on three random regions.

### Immunofluorescence staining

The cells were fixed using 4% paraformaldehyde for 20 minutes. 0.5% Triton X-100 was used for permeabilization for 10 minutes and then blocked with 5% BSA (bovine serum albumin) for 1 hour. Next, it was incubated with the RASA1 primary antibody (1:100, ZEN Bio, China) overnight at 4 °C. The secondary antibody conjugated to Dylight (1:200, Abbkine, China) was then added for incubation for 1 hour at room temperature. Finally, the nucleus was stained by adding Hoechst solution (Beyotime, China), and the stained cells were photographed.

### Western blots

We prepared cell lysates using RIPA buffer (Thermo Scientific). Then, a BCA Protein Assay Kit (Pierce, Thermo Scientific) was used to determine the concentration of the extracted protein. It was separated by 8% SDS-PAGE electrophoresis and transferred to a PVDF membrane (Millipore). It was then blocked with 5% skim milk for 1 hour at room temperature and incubated overnight at 4 °C with primary anti-RASA1 (1:1000, ZEN Bio, China). The HRP-labeled secondary antibody (CST) was then incubated for 1 hour at room temperature. Finally, chemiluminescence methods were used for protein detection. The internal reference used anti-GAPDH antibody (1:1,000, Affinity, USA).

### Immunohistochemical staining

Tissue immunohistochemistry (IHC) staining was performed on formalin-fixed, paraffin-embedded tissue sections using an IHC test kit (Kangwei shiji Biotechnology). Paraffin sections were deparaffinized, antigen was repaired with sodium citrate buffer, and then incubated with 3% hydrogen peroxide for 15 minutes at room temperature to block endogenous peroxidase and then blocked with 10% goat serum for 30 minutes. The RASA1 antibody (1:100, ZEN Bio, China) was incubated overnight at 4 °C in a humid chamber and then incubated with the secondary antibody for 1 hour at room temperature. The color reaction was carried out by DAB, and once browned, the reaction was terminated by immersing the tissue sections in PBS. Tissue sections were counterstained with hematoxylin, dehydrated and fixed in gradient ethanol. IHC staining is based on the percentage of positively stained tumor cells, 0 means less than 5% of tumor cells stain positive, 1 means 5-30%, 2 means 31-50%, 3 means 51-80%, 4 means > 80% positive staining. The expression of RASA1 in the TNBC specimen was divided into a low expression group (0-1 points) and a high expression group (2-4 points) according to the score.

### Animal experiments

Breast cancer cells (5 x 10^6^) were injected subcutaneously into female nude mice. The mice were sacrificed 4 weeks later and the tumor was excised and the tumor weight was measured. An animal model of lung metastasis was constructed by injecting breast cancer cells (1 x 10^6^) into nude mice through the tail vein. Detection of lung metastases was performed under anesthesia by a small animal in vivo imaging system (Bruke MI, USA). After 6 weeks, the mice were sacrificed and the lungs were removed and the number of lung metastatic nodules was counted. It was confirmed by microscopy after staining with hematoxylin and eosin (HE). Animal experiments were approved by the Sun Yat-sen University Cancer Center Animal Experimental Ethics Committee and conducted at the Sun Yat-sen University Cancer Center Animal Experimental Center.

### RNA immunoprecipitation (RIP) assay

For the GFP-MS2-RIP assay, MS2bs-circAHNAK1, MS2bs-circAHNAK1mt or MS2bs-Rluc and MS2bp-GFP were used to co-transfect cells. After 48 hours, RIP assays were performed using the Magna RIP RNA-Binding Protein Immunoprecipitation Kit (Millipore, USA) to determine if the RNA complex contained circAHNAK1 and its potentially binding miR-421. The immunoprecipitated RNA was subsequently quantitatively analyzed by qRT-PCR. For Ago2-RIP assay, anti-Ago2 antibody (Millipore) was used for immunoprecipitation. RNA complex contained miR-421 and its potential binding circAHNAK1 or RASA1 mRNA were then purified and quantified by qRT-PCR.

### Luciferase reporter assay

The circAHNAK1 sequence containing the miR-421 binding site (AGGACCCAAGGUGGA-UGUUGAAG) was synthesized and cloned into the luciferase vector pGL3 (Promega). Mutant miR-421 binding sites were performed using the Fast SiteDirected Mutagenesis Kit (TIANGEN, China). The wild type and mutant 3′UTR (UUUCUCUUGCUGGAACUGUUGAA) of RASA1 containing the miR-421 binding site was synthesized and cloned into the luciferase vector. The vectors constructed above were then co-transfected with the miR-421 mimics or inhibitor. Luciferase activity was detected by the dual luciferase reporter kit (Promega) 48 hours after transfection.

### Statistical analysis

Statistical analysis was performed using SPSS 21.0 statistical software. Measurement data were determined statistically by Student’s t-test (two-tailed) (P < 0.05), while count data was assessed by chi-square test. Differences in survival were assessed using Kaplan-Meier plots and log-rank tests.

## Supplementary Material

Supplementary Figure 1

Supplementary Table 1

## References

[1] Foulkes WD, Smith IE, Reis-Filho JS. Triple-negative breast cancer. N Engl J Med. 2010; 363:1938–48. 10.1056/NEJMra1001389 21067385

[2] Waks AG, Winer EP. Breast Cancer Treatment: A Review. JAMA. 2019; 321:288–300. 10.1001/jama.2018.19323 30667505

[3] Bianchini G, Balko JM, Mayer IA, Sanders ME, Gianni L. Triple-negative breast cancer: challenges and opportunities of a heterogeneous disease. Nat Rev Clin Oncol. 2016; 13:674–90. 10.1038/nrclinonc.2016.66 27184417 PMC5461122

[4] Vo JN, Cieslik M, Zhang Y, Shukla S, Xiao L, Zhang Y, Wu YM, Dhanasekaran SM, Engelke CG, Cao X, Robinson DR, Nesvizhskii AI, Chinnaiyan AM. The Landscape of Circular RNA in Cancer. Cell. 2019; 176:869–881.e13. 10.1016/j.cell.2018.12.021 30735636 PMC6601354

[5] Szabo L, Salzman J. Detecting circular RNAs: bioinformatic and experimental challenges. Nat Rev Genet. 2016; 17:679–92. 10.1038/nrg.2016.114 27739534 PMC5565156

[6] Han D, Li J, Wang H, Su X, Hou J, Gu Y, Qian C, Lin Y, Liu X, Huang M, Li N, Zhou W, Yu Y, Cao X. Circular RNA circMTO1 acts as the sponge of microRNA-9 to suppress hepatocellular carcinoma progression. Hepatology. 2017; 66:1151–64. 10.1002/hep.29270 28520103

[7] Zheng Q, Bao C, Guo W, Li S, Chen J, Chen B, Luo Y, Lyu D, Li Y, Shi G, Liang L, Gu J, He X, Huang S. Circular RNA profiling reveals an abundant circHIPK3 that regulates cell growth by sponging multiple miRNAs. Nat Commun. 2016; 7:11215. 10.1038/ncomms11215 27050392 PMC4823868

[8] Tang H, Huang X, Wang J, Yang L, Kong Y, Gao G, Zhang L, Chen ZS, Xie X. circKIF4A acts as a prognostic factor and mediator to regulate the progression of triple-negative breast cancer. Mol Cancer. 2019; 18:23. 10.1186/s12943-019-0946-x 30744636 PMC6369546

[9] Xu JZ, Shao CC, Wang XJ, Zhao X, Chen JQ, Ouyang YX, Feng J, Zhang F, Huang WH, Ying Q, Chen CF, Wei XL, Dong HY, et al. circTADA2As suppress breast cancer progression and metastasis via targeting miR-203a-3p/SOCS3 axis. Cell Death Dis. 2019; 10:175. 10.1038/s41419-019-1382-y 30787278 PMC6382814

[10] Yang R, Xing L, Zheng X, Sun Y, Wang X, Chen J. The circRNA circAGFG1 acts as a sponge of miR-195-5p to promote triple-negative breast cancer progression through regulating CCNE1 expression. Mol Cancer. 2019; 18:4. 10.1186/s12943-018-0933-7 30621700 PMC6325825

[11] Chen B, Wei W, Huang X, Xie X, Kong Y, Dai D, Yang L, Wang J, Tang H, Xie X. circEPSTI1 as a Prognostic Marker and Mediator of Triple-Negative Breast Cancer Progression. Theranostics. 2018; 8:4003–15. 10.7150/thno.24106 30083277 PMC6071524

[12] Qiu M, Xia W, Chen R, Wang S, Xu Y, Ma Z, Xu W, Zhang E, Wang J, Fang T, Hu J, Dong G, Yin R, et al. The Circular RNA circPRKCI Promotes Tumor Growth in Lung Adenocarcinoma. Cancer Res. 2018; 78:2839–51. 10.1158/0008-5472.CAN-17-2808 29588350

[13] Li Q, Pan X, Zhu D, Deng Z, Jiang R, Wang X. Circular RNA MAT2B promotes glycolysis and malignancy of hepatocellular carcinoma via the miR-338-3p/PKM2 axis under hypoxic stress. Hepatology. 2019; 70:1298–316. 10.1002/hep.30671 31004447

[14] Tian XP, Huang WJ, Huang HQ, Liu YH, Wang L, Zhang X, Lin TY, Rao HL, Li M, Liu F, Zhang F, Zhong LY, Liang L, et al. Prognostic and predictive value of a microRNA signature in adults with T-cell lymphoblastic lymphoma. Leukemia. 2019; 33:2454–65. 10.1038/s41375-019-0466-0 30953029

[15] Zhang Y, Gong W, Dai S, Huang G, Shen X, Gao M, Xu Z, Zeng Y, He F. Downregulation of human farnesoid X receptor by miR-421 promotes proliferation and migration of hepatocellular carcinoma cells. Mol Cancer Res. 2012; 10:516–22. 10.1158/1541-7786.MCR-11-0473 22446874

[16] Jiang Z, Guo J, Xiao B, Miao Y, Huang R, Li D, Zhang Y. Increased expression of miR-421 in human gastric carcinoma and its clinical association. J Gastroenterol. 2010; 45:17–23. 10.1007/s00535-009-0135-6 19802518

[17] Hu TB, Chen HS, Cao MQ, Guo FD, Cheng XY, Han ZB, Li MQ. MicroRNA-421 inhibits caspase-10 expression and promotes breast cancer progression. Neoplasma. 2018; 65:49–54. 10.4149/neo_2018_170306N159 29322788

[18] Li Y, Cui X, Li Y, Zhang T, Li S. Upregulated expression of miR-421 is associated with poor prognosis in non-small-cell lung cancer. Cancer Manag Res. 2018; 10:2627–33. 10.2147/CMAR.S167432 30147363 PMC6095112

[19] Bueno RC, Canevari RA, Villacis RA, Domingues MA, Caldeira JR, Rocha RM, Drigo SA, Rogatto SR. ATM down-regulation is associated with poor prognosis in sporadic breast carcinomas. Ann Oncol. 2014; 25:69–75. 10.1093/annonc/mdt421 24285016

[20] Yin Y, Xu L, Chang Y, Zeng T, Chen X, Wang A, Groth J, Foo WC, Liang C, Hu H, Huang J. N-Myc promotes therapeutic resistance development of neuroendocrine prostate cancer by differentially regulating miR-421/ATM pathway. Mol Cancer. 2019; 18:11. 10.1186/s12943-019-0941-2 30657058 PMC6337850

[21] Suárez-Cabrera C, Quintana RM, Bravo A, Casanova ML, Page A, Alameda JP, Paramio JM, Maroto A, Salamanca J, Dupuy AJ, Ramírez A, Navarro M. A Transposon-based Analysis Reveals *RASA1* Is Involved in Triple-Negative Breast Cancer. Cancer Res. 2017; 77:1357–68. 10.1158/0008-5472.CAN-16-1586 28108518

[22] Kent OA, Mendell JT, Rottapel R. Transcriptional Regulation of miR-31 by Oncogenic KRAS Mediates Metastatic Phenotypes by Repressing RASA1. Mol Cancer Res. 2016; 14:267–77. 10.1158/1541-7786.MCR-15-0456 26747707 PMC4794362

[23] Sun D, Wang C, Long S, Ma Y, Guo Y, Huang Z, Chen X, Zhang C, Chen J, Zhang J. C/EBP-β-activated microRNA-223 promotes tumour growth through targeting RASA1 in human colorectal cancer. Br J Cancer. 2015; 112:1491–500. 10.1038/bjc.2015.107 25867276 PMC4453668

[24] Hayashi T, Desmeules P, Smith RS, Drilon A, Somwar R, Ladanyi M. *RASA1* and *NF1* are Preferentially Co-Mutated and Define A Distinct Genetic Subset of Smoking-Associated Non-Small Cell Lung Carcinomas Sensitive to MEK Inhibition. Clin Cancer Res. 2018; 24:1436–47. 10.1158/1078-0432.CCR-17-2343 29127119 PMC6440215

[25] Du C, Weng X, Hu W, Lv Z, Xiao H, Ding C, Gyabaah OA, Xie H, Zhou L, Wu J, Zheng S. Hypoxia-inducible MiR-182 promotes angiogenesis by targeting RASA1 in hepatocellular carcinoma. J Exp Clin Cancer Res. 2015; 34:67. 10.1186/s13046-015-0182-1 26126858 PMC4493986

