# Supplementary material for: CircAHNAK1 inhibits proliferation and metastasis of triple-negative breast cancer by modulating miR-421 and RASA1
Source: Aging (Albany NY). 2019 Dec 19;11(24):12043–56. doi: 10.18632/aging.102539 (PMC6949091; doi:10.18632/aging.102539)
Supplement: Supplementary Figure 1 [file aging-11-102539-s002..pdf]

SUPPLEMENTARY FIGURE

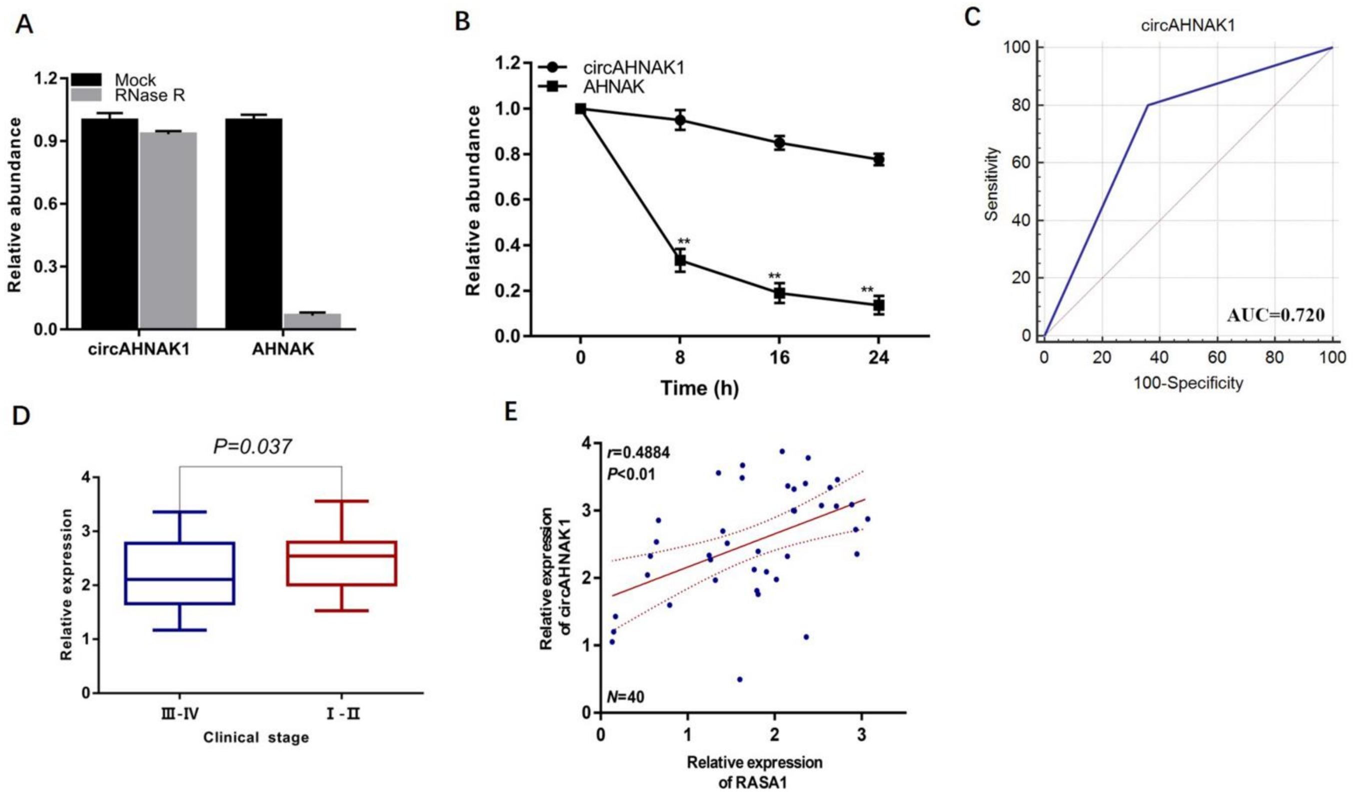

Supplementary Figure 1. Characterization of circular RNA CircAHNAK1 in TNBC. (**A**) Analysis of abundance of circAHNAK1 and AHNAK mRNA in MDA-MB-231 cells treated with RNase R by qRT-PCR; (**B**) qRT-PCR analysis of the abundance of circAHNAK1 and AHNAK mRNA after actinomycin D treatment of MDA-MB-231 cells; (**C**) ROC analysis suggested that circAHNAK1 can be used as a diagnostic indicator for distinguishing between TNBC and normal breast tissue; (**D**) circRNA is significantly down-regulated in stage III–IV TNBC tissues compared to stage I–II; (**E**) Correlation analysis showed a significant linear correlation between circAHNAK1 and RASA1.
