# Supplementary material for: CircAHNAK1 inhibits proliferation and metastasis of triple-negative breast cancer by modulating miR-421 and RASA1
Source: Aging (Albany NY). 2019 Dec 19;11(24):12043–56. doi: 10.18632/aging.102539 (PMC6949091; doi:10.18632/aging.102539)
Supplement: Supplementary Table 1 [file aging-11-102539-s001..pdf]

SUPPLEMENTARY TABLE

Supplement Table 1. Primer sequences for qRT-PCR used in this study.

Construct	Primers	Sequence (5′ - 3′)
**circAHNAK1**	Forward	CATGCCTGATGTGGACCTGA
	Reverse	CAGTCTGGGCCTTGAACCTC
**18S**	Forward	TTAATTCCGATAACGAACGAGA
	Reverse	CGCTGAGCCAGTCAGTGTAG
**β-actin**	Forward	AGCGAGCATCCCCCAAAGTT
	Reverse	GGGCACGAAGGCTCATCATT
**GAPDH**	Forward	GGAGCGAGATCCCTCCAAAAT
	Reverse	GGCTGTTGTCATACTTCTCATGG
**RASA1**	Forward	TGTAGTTGAGGTCAATGAAGGG
	Reverse	TGGCCACCTGTTCCTCCTCGTATT
